# Correlation between AI-based CT organ features and normal lung dose in adjuvant radiotherapy following breast-conserving surgery: a multicenter prospective study

**DOI:** 10.1186/s12885-023-11554-2

**Published:** 2023-11-09

**Authors:** Li Ma, Yongjing Yang, Jiabao Ma, Li Mao, Xiuli Li, Lingling Feng, Muyasha Abulimiti, Xiaoyong Xiang, Fangmeng Fu, Yutong Tan, Wenjue Zhang, Ye-Xiong Li, Jing Jin, Ning Li

**Affiliations:** 1 National Cancer Center/National Clinical Research Center for Cancer/Cancer Hospital & Shenzhen Hospital, Chinese Academy of Medical Sciences and Peking Union Medical College, Shenzhen, 518116 China; 2 Department of Radiation Oncology, Jilin Cancer Hospital, Changchun, Jilin 130012 China; 3 Department of Radiation Oncology, Sichuan Cancer Hospital & Research Institute, No. 55, the 4Th Section, Renmin South Road, Chengdu, 610041 China; 4 AI Lab, Deepwise Healthcare, Beijing, 100080 People’s Republic of China; 5 Department of Radiation Oncology, Cancer Hospital, Chinese Academy of Medical Sciences, Peking Union Medical College, Beijing, China; 6 Shanxi Province Cancer Hospital/Shanxi Hospital Affiliated to Cancer Hospital, Chinese Academy of Medical Sciences/Cancer Hospital Affiliated to Shanxi Medical University, Jinzhong, China

**Keywords:** Breast cancer, Adjuvant radiotherapy, Lung V20, Deep learning

## Abstract

**Background:**

Radiation pneumonitis (RP) is one of the common side effects after adjuvant radiotherapy in breast cancer. Irradiation dose to normal lung was related to RP. We aimed to propose an organ features based on deep learning (DL) model and to evaluate the correlation between normal lung dose and organ features.

**Methods:**

Patients with pathology-confirmed invasive breast cancer treated with adjuvant radiotherapy following breast-conserving surgery in four centers were included. From 2019 to 2020, a total of 230 patients from four nationwide centers in China were screened, of whom 208 were enrolled for DL modeling, and 22 patients from another three centers formed the external testing cohort. The subset of the internal testing cohort (*n* = 42) formed the internal correlation testing cohort for correlation analysis. The outline of the ipsilateral breast was marked with a lead wire before the scanning. Then, a DL model based on the High-Resolution Net was developed to detect the lead wire marker in each slice of the CT images automatically, and an in-house model was applied to segment the ipsilateral lung region. The mean and standard deviation of the distance error, the average precision, and average recall were used to measure the performance of the lead wire marker detection model. Based on these DL model results, we proposed an organ feature, and the Pearson correlation coefficient was calculated between the proposed organ feature and ipsilateral lung volume receiving 20 Gray (Gy) or more (V20).

**Results:**

For the lead wire marker detection model, the mean and standard deviation of the distance error, AP (5 mm) and AR (5 mm) reached 3.415 ± 4.529, 0.860, 0.883, and 4.189 ± 8.390, 0.848, 0.830 in the internal testing cohort and external testing cohort, respectively. The proposed organ feature calculated from the detected marker correlated with ipsilateral lung V20 (Pearson correlation coefficient, 0.542 with *p* < 0.001 in the internal correlation testing cohort and 0.554 with *p* = 0.008 in the external testing cohort).

**Conclusions:**

The proposed artificial Intelligence-based CT organ feature was correlated with normal lung dose in adjuvant radiotherapy following breast-conserving surgery in patients with invasive breast cancer.

**Trial registration:**

NCT05609058 (08/11/2022).

## Background

Breast cancer remains the most common cancer in women and one of the leading causes of cancer-related death [1]. Adjuvant radiotherapy is the essential treatment strategy for early breast cancer patients who receive breast-conserving surgery, which decreases local recurrence and improves overall survival [2]. However, radiation pneumonitis (RP) often occurs during and after radiotherapy. The incidence of clinical RP is reported to be 14% to 19.56% [3–5], which negatively affects patients’ quality of life. In the early identification of high-risk patients, clinicians can take early intervention measures to reduce the incidence of RP.

The fundamentally effective approach to decrease the incidence of RP is to limit the lung radiation dose. Several parameters have been reported to be involved in the process of RP, including clinical factors and dosimetric factors [3, 4, 6]. Dosimetric parameters, including ipsilateral lung volume receiving 20 Gray (Gy) or more (V20), V30, the dose at least delivered to 25% of the volume of the ipsilateral lung and mean lung dose, have been demonstrated to be related to RP in breast cancer [4, 6].

We hypothesized that patient-specific body geometry reflects the lung irradiation dose. Thus, our study attempts to construct an organ feature to reflect specific geometry using body markers. However, labeling markers manually is time-consuming. In recent years, deep learning (DL) has shown excellent performance in medical image segmentation, recognition, and classification [7–13]. However, using a DL model in body marker detection that guided radiotherapy has not yet been reported. In our study, we attempts to construct a DL model for marker detection and propose the normal lung dose correlated organ features in breast cancer in the setting of adjuvant radiotherapy.

## Methods

### Patients

Patients in the prospective study (ClinicalTrial.gov NCT05609058, 08/11/2022) who underwent breast-conserving surgery and sentinel node biopsy or axillary dissection, and pathology confirmed invasive breast cancer with no residual microscopic disease from four nationwide centers in China were included.

Between Jan 2019 and Oct 2020, a total of 230 patients from four nationwide centers in China were screened, of whom 208 in one center were enrolled for DL modeling and were divided into the training cohort (*n* = 128), the validation cohort (*n* = 20), and the internal testing cohort (*n* = 60), and 22 in other three centers were used as an external testing cohort. Patients with invasive breast cancer without supra/infraclavicular nodal irradiation (*n* = 42) formed the internal correlation testing cohort which was a subset of the internal testing cohort and was used to validate the performance of the automatically calculated organ features.

### Position and radiation planning

All patients were immobilized on a breast bracket (CIVCO Medical Solutions, Orange City, IA, USA) for CT simulation in the supine position. The outline of the ipsilateral breast was marked with a lead wire by physical examination before the CT scan. A treatment planning CT scan was performed with 5.0 mm thickness slices from the Atlas (C1) to the second lumbar vertebra (L2) level to cover the whole breast using a 16-slice CT scanner (GE Discovery RT, GE Healthcare, Chicago, IL, USA).

The clinical target volume (CTV) was defined as the whole breast according to the marked outline and the fascia of the pectoralis major. The tumor bed target volume for the boost was determined according to surgical changes and silver clips. The planning target volume (PTV) boost and PTV were formed by extending a 5.0 mm margin from CTV and CTV boost, respectively, within 5.0 mm inside of the body outline. Patients who had more than 4 positive axillary nodes or one to three positive nodes with risk factors received additional supra/infraclavicular nodal irradiation. The radiation dose was delivered by intensity modulation radiation therapy with tangential beams. PTV was given 50 Gy in 25 fractions. PTVboost was given 60 Gy in 25 or 30 fractions.

One attending radiation oncologist (L.M.) marked lead wire on CT images using the Deepwise Research Platform (Deepwise Inc., Beijing, China, http://label.deepwise.com) and a senior radiation oncologist (N.L.) reviewed all the markers.

### The automatic segmentation of the ipsilateral lung

The pulmonary lobe region of the CT images was segmented by the previously trained in-house segmentation model first. The segmentation model was a 2.5D U-Net-based model that was constructed with a 2D encoder and a 3D decoder. The 2D encoder had the same structure as ResNet34, and the pre-trained weight on ImageNet was used to initialize the parameters. The model was trained using 1434 CT images with 58,019 slices and reached an average Dice of 92.8.

After the region of the pulmonary lobes was segmented, the area of the ipsilateral lung was calculated and documented.

### The lead wire marker detection model

**Fig. 1:**
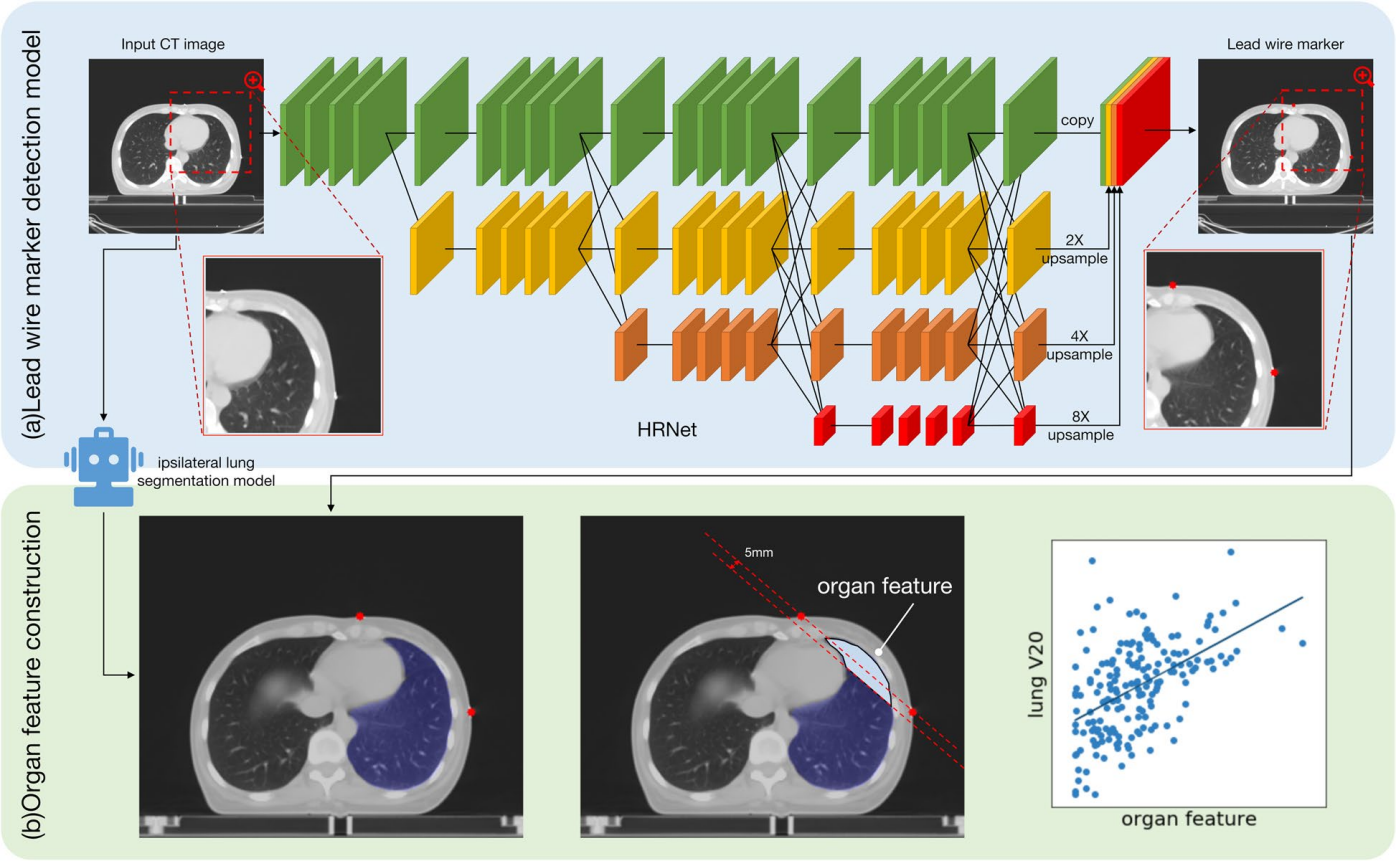
The overall workflow of our study. **a** The structure of the lead wire marker detection model. The model includes four stages, and at the final stage, the feature maps were concatenated together to predict the lead wire marker location. **b** Illustration of the proposed organ feature construction procedure

In this study, we developed a DL model to detect the lead wire marker on CT images. The lead wire marker detection model was built based on HRNet [14]. The model included four stages, in which parallel multiresolution convolutions were used. Multiresolution fusion was performed between the stages. In the final stage, the branches of the different resolutions were combined to generate two heatmaps. The location of the maximum values of the two heatmaps matched the two lead wire markers on each CT slice. This procedure is shown in Fig. 1.

Before reaching the network, the CT images were clipped into [-140, 210] and then normalized to [0, 1]. For the prediction of each slice, the target slice and its adjacent two slices were combined to form three channels. The Adam optimizer was used to minimize the mean square error loss, with an initial learning rate of 0.0001. The model was trained on the training cohort for 2000 epochs, and the model that reached the lowest loss in the validation cohort was used.

After the model was trained, the mean and standard deviation of the distance between the location of the predicted marker and the labeled marker were measured, and the average precision (AP) and average recall (AR) were calculated.

### Construction of lung dose-related organ features

The lung dose-related organ feature was constructed by estimating the volume of the lung in the tangential beams. For each slice, the two detected lead wire markers were used to determine the margin of the exposed lung region. Then, the margin was moved 5.0 mm inward according to the PTV outline. The segmented area of the ipsilateral lung region outside the exposed margin was calculated, and the sum of the calculated areas for each slice was then divided by the total ipsilateral lung region a and used as the proposed organ feature. Figure 1b illustrates this procedure.

### Statistical analysis

All statistical results were calculated in Python and R (version 3.6.0; https://www.r-project.org/). The correlation between the proposed organ feature and ipsilateral lung V20 was assessed by the Pearson correlation coefficient. A Pearson correlation coefficient between 0 and 0.3 (or between 0 and -0.3) indicates a weak relationship between the two variables. A Pearson correlation coefficient between 0.3 and 0.5 (or between -0.3 and -0.5) indicates a moderate strength relationship between the two variables. A Pearson correlation coefficient between 0.5 and 1 (or between -0.5 and 1) indicates a correlation between the two variables [15]. A *p-*value of < 0.05 was considered to indicate a significant difference.

## Results

### Enrollment and clinical characteristics

**Fig. 2:**
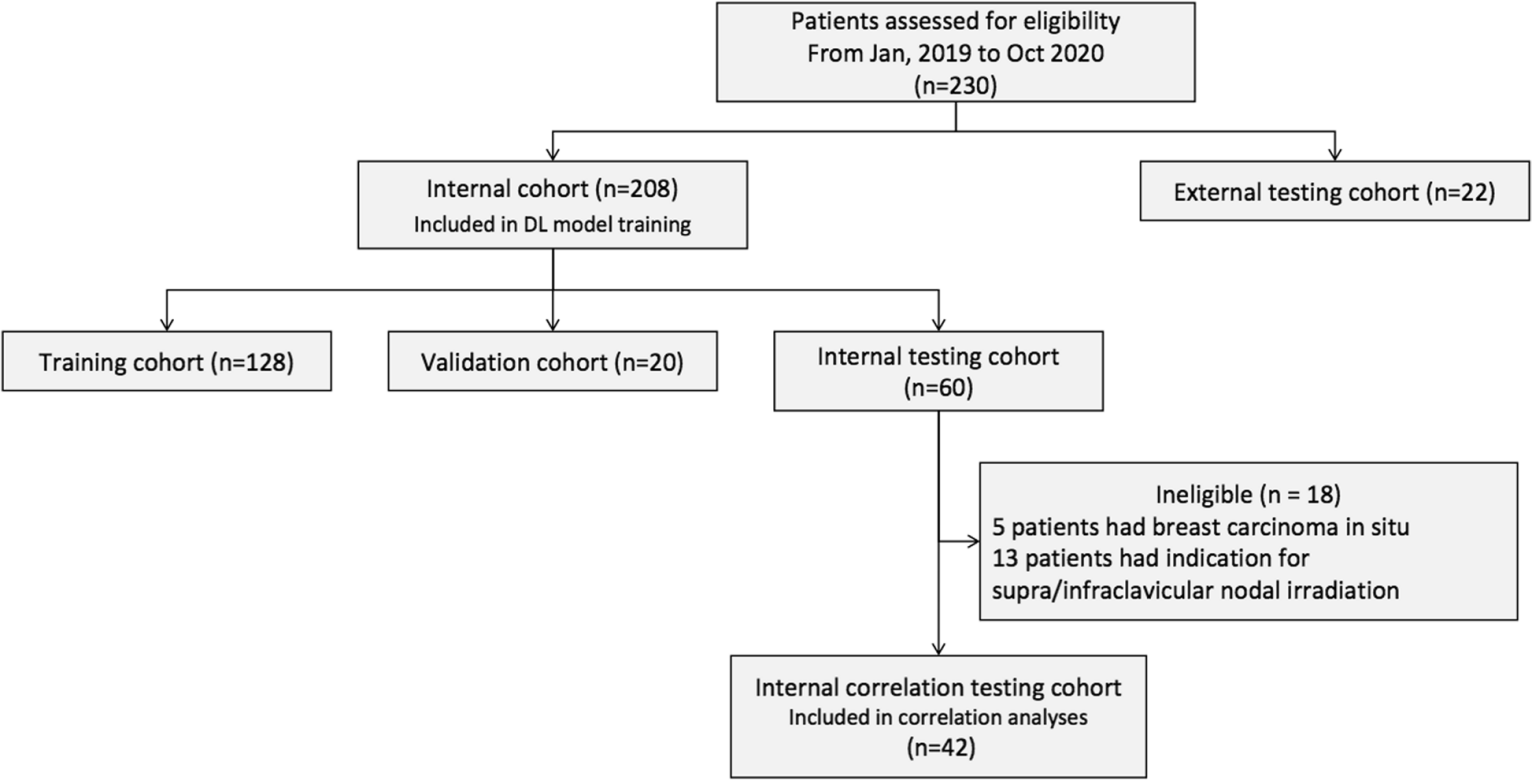
The flowchart of the inclusion and exclusion pipeline of our study

Details of the patient’s baseline characteristics are shown in Table 1, and the pipeline is shown in Fig. 2. Left-sided breast cancer accounted for 51.4% (*n* = 107). The median volumes of PTV and PTV-boost were 666.7 (range 214.6–1841) cm^3^ and 96.83 (range 28.49–260.13) cm^3^, respectively. The median ipsilateral lung V20 was 17.54 (range 7.75–29.08) %.

**Table 1:** Baseline characteristics

	Internal correlation cohort (*n* = 208)	External testing cohort (*n* = 22)
Age, years		
Median	47	50
Range	27–68	32–64
Sex		
Female	208 (100%)	22 (100%)
Tumor location		
Left	98 (47.1%)	9 (40.9%)
Right	110 (52.9%)	13 (59%)
PTV, cm^3^		
Median	667	623.61
Range	214.6–1841	331.46–1303.6
PTVboost, cm^3^		
Median	101.89	62.16
Range	37.4–260.13	28.49–165.48
Ipsilateral lung V20, %		
Median	17.41	18.04
Range	7.75–29.08	8.26–24.66

*PTV* Planning target volume, *ipsilateral lung V20* Ipsilateral lung volume receiving 20 Gray or more

### The performance of the lead wire marker detection model

Table 2 shows the performance of the lead wire marker detection model. In the internal testing cohort, the mean and standard deviation of the distance between the predicted location of the lead wire marker and the labeled lead wire marker was 3.415 ± 4.529. In the external testing cohort, the distance was 4.189 ± 8.390. The AP and AR at a distance tolerance of 5 mm were 0.860 and 0.883, respectively, in the internal testing cohort and reached 0.848 and 0.830, respectively, in the external testing cohort. When the tolerance was 10 mm, the AP and AR on both testing cohorts were greater than 0.9.

**Table 2:** The performance of the lead wire marker detection model

Dataset	dist (mm)	AP@5 mm	AP@10 mm	AR@5 mm	AR@10 mm
Internal testing cohort	3.415 ± 4.529	0.860	0.966	0.883	0.991
External testing cohort	4.189 ± 8.390	0.848	0.928	0.830	0.909

### The correlation between the proposed organ feature and ipsilateral lung V20

The proposed organ feature that was calculated by the automatically detected marker correlated with ipsilateral lung V20 (Pearson correlation coefficient, 0.542 with *p* < 0.001 in the internal correlation testing cohort and 0.554 with *p* = 0.008 in the external testing cohort).

**Fig. 3:**
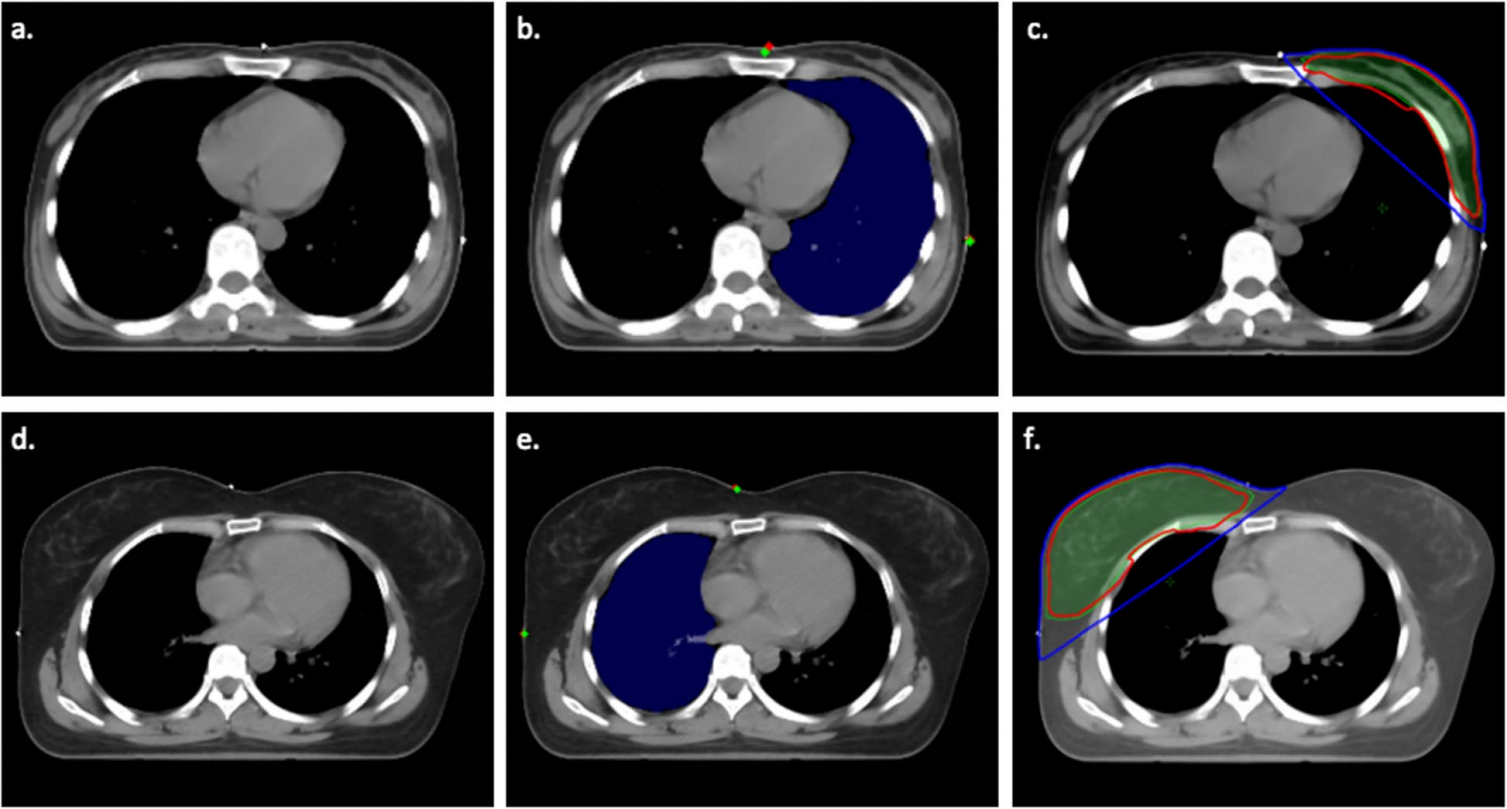
Lung segmentation, lead wire marker detection results and radiation dose distribution. The blue mask was the automatic segmented ipsilateral lung region, the red points were the manually annotated lead wire marker, and the green points were the lead wire marker detected by the proposed DL model. The green area represented the planning target volume. The red line and blue line represented 50 Gy and 20 Gy isodose line, respectively. **a**, **b** and **c** Show the results of a 41-year-old female, with an organ feature value of 0.204 and lung V20 of 19.86. **c**, **d** and **e** Show the results of a 30-year-old female, with an organ feature value of 0.010 and lung V20 of 12.76

As for the proposed organ feature that was calculated by the manually labeled lead wire marker, a similar result can be found (Pearson correlation coefficient was 0.560 with *p* < 0.001 in the internal correlation testing cohort and 0.613 with *p* < 0.001 in the external testing cohort). The Pearson correlation coefficient was higher than the automatically detected marker-based organ feature, but no significance was found (paired t-test, *p* = 0.921 in the internal correlation testing cohort and 0.586 in the external testing cohort) (Table 3). Figure 3 showed two example cases with different chest geometry that had a relationship with differences in lung radiation dose.

**Table 3:** The correlation between the proposed organ feature and ipsilateral lung V20

Dataset	Case number	Pearson correlation	*p* value
Calculated by labeled lead wire marker			
Internal correlation testing cohort	42	0.560	< 0.001
External testing cohort	22	0.613	< 0.001
Calculated by the detected lead wire marker			
Internal correlation testing cohort	42	0.542	< 0.001
External testing cohort	22	0.554	0.008

## Discussion

In this study, we developed a deep-learning-based lead wire marker detection model. The model resulted in high AP and AR in both the internal and external testing cohorts. The proposed organ feature built upon the automatically detected lead wire marker correlated with the lung V20 in patients with early invasive breast cancer who received breast-conserving surgery.

The lung is a critical dose-limiting organ during adjuvant radiotherapy in breast cancer, with radiation pneumonitis being a common complication. Ipsilateral lung V20 has been widely used as a critical dosimetric indicator for RP in breast cancer. It is reported to be positively related to the incidence of RP [4, 16–20]. According to receiver operating characteristic curve analysis, Lind and colleagues suggested a significant correlation between ipsilateral lung V20 and clinical RP (*p* = 0.008) and radiological RP (*p* = 0.009) in breast cancer [16]. For V20, using a cutoff point of 20.2%, clinical RP could be predicted with an accuracy of 88.7%, a sensitivity of 83.3%, and a specificity of 89.6% [17]. Consistent with these results, a lower incidence of RP was found when ipsilateral lung V20 ≤ 20% as compared to V20 > 20% (12.5% vs. 28.4%, respectively) [4]. Ozgen et al. also revealed that V20 (cutoff value of 23%) played a significant role in predicting RP (*p* = 0.017) [18]. In the retrospective study of Koreans, ipsilateral lung V20 was reported to be significantly associated with RP (*p* = 0.018) [20]. Similarly, our study aimed to develop a model that could reflect the normal lung dose. Ipsilateral lung V20 was the most widely used predictor of RP and thus was selected as a pivotal endpoint parameter.

DL in predicting irradiation dose has been explored in various tumors and has shown promising results [12, 13, 21–24]. It has been applied in many tasks for medical imaging analysis, including recognition of disease from normal patterns, classification of malignant and benign lesions, prediction of tumor prognosis, and radiation dose distribution. In the context of lead wire detection, while certain conventional techniques may show promise due to the well-defined shape and high contrast of the wires, they may prove inadequate when confronted with variations in wire shape resulting from tilting. Furthermore, these methods may also be prone to confusion with other objects that bear a similar appearance. Hence, in this study, a DL model has been employed for lead wire detection.

The relative variability of the clinical target volume of breast cancer radiotherapy is small, and radiotherapy is mainly delivered by tangent beams. Therefore, the radiation dose to the ipsilateral lung may be correlated with the patient's chest geometry. In breast cancer, a dose prediction model was developed based on DL and was demonstrated to accurately predict patient-specific doses. Ahn et al. conducted a study on the prediction of dose volume histogram using 50 volumetric modulated arc therapy plans of left-sided breast cancer patients with a prescription of 4320 cGy in 16 fractions. The mean absolute error and one standard deviation between the clinical and DL dose prediction models were − 1.16 ± 2.58% for the ipsilateral lung [24]. Similarly, Bakx et al. compared the performance of two dose prediction models by studying 105 left-sided breast cancers. Each patient had a prescribed dose of 4005 cGy in 15 fractions. They confirmed a small difference (*p* < 0.05) between predicted plans and clinical plans [13]. Another study by Hedden et al. constructed two-dimensional and three-dimensional DL models and demonstrated comparable dose distributions with clinical plans. In this study, the patients were treated in 16 fractions with a prescribed total dose of 42.56 Gy [23]. In consistent with previous studies, the purpose of our study was to construct a model that could reflect the normal lung dose. We developed an organ feature based on DL and found a correlation between lung V20 and image features with a Pearson correlation coefficient > 0.50. Our study suggests that patient-specific body geometry could reflect lung irradiation dose.

Importantly, the aforementioned three studies exclusively focused on left-sided breast cancers, which introduces potential sampling bias concerns. In contrast, our study encompassed patients irrespective of the lesion location, ensuring a more comprehensive evaluation. Furthermore, irradiation to the whole breast with a boost to tumorbed is the standard treatment for invasive breast cancer who received breast-conserving surgery. However, the previous studies only evaluated models without a boost dose. Different from these studies, patients tested in our model were prescribed a 50 Gy to whole breast and a boost of 10 Gy to tumorbed which were more fit for clinical practice. Besides, the mentioned studies consisted of single-center data. There may be confounding factors. To our knowledge, our study was the first multicenter study with real-world external validation, and the sample size was larger than previously reported. A total of 64 patients formed the testing cohort, including 22 patients in the external testing cohort. In addition, previous articles focused on developing different prediction models to help reduce the time required to produce clinical plans. Our investigation attempted to identify a personal geometric feature that could predict lung V20 and further select more appropriate irradiation techniques for individual patients.

Our study had several limitations. First, this pilot study only evaluated the lung irradiation dose. Other organs at risk and more dose parameters could be further investigated. Second, only the correlation coefficient was analyzed in this study. We are working on the multiparameter prediction model of the lung irradiation dose.

## Conclusions

AI can be used to predict normal lung dose in adjuvant radiotherapy following breast-conserving surgery for invasive breast cancer. Early identification of high lung V20 could be a reminder of select more appropriate irradiation techniques in these patients, like deep inspiration breath holding, accelerated partial breast irradiation, etc.

## Data Availability

The datasets generated and/or analysed during the current study are not publicly available due protection of personal data but are available from the corresponding author on reasonable request.

## Abbreviations

DL: Deep learning
HRNet: High-Resolution Net
AP: Average precision
AR: Average recall
Gy: Gray
Lung V20: Lung volume receiving 20 Gray (Gy) or more
AI: Artificial Intelligence
RP: Radiation pneumonitis
CTV: Clinical target volume
PTV: Planning target volume
